# Meeting report of the International Policy Dialogue on HIV/AIDS and Disability

**DOI:** 10.1186/1758-2652-12-27

**Published:** 2009-11-09

**Authors:** Sharon Peake

**Affiliations:** 1 International Health Division, International Affairs Directorate, Health Canada, Canada

## Abstract

As part of a partnership arrangement with the Joint United Nations Programme on HIV/AIDS and the Public Health Agency of Canada, Health Canada hosted an International Policy Dialogue on HIV/AIDS and Disability from 11 to 13 March 2009 in Ottawa, Canada. The dialogue provided a forum for stakeholders from governments, academia, and non-governmental and multilateral organizations to explore the issues and evidence related to HIV/AIDS and disability, and to chart a way forward in terms of policy and programme development. This meeting report outlines the participants, objectives and high-level outcomes.

## Introduction

Health Canada, as part of a partnership arrangement with the Joint United Nations Programme on HIV/AIDS (UNAIDS) and the Public Health Agency of Canada, hosted an International Policy Dialogue on HIV/AIDS and Disability from 11 to 13 March 2009 in Ottawa, Canada. The objectives of the dialogue were: to explore the place of disability in the changing nature of the global HIV/AIDS epidemic and the required response; to share and learn from participants' experiences; and to build and reinforce the partnerships needed to sustain a comprehensive global response to HIV/AIDS, which includes issues related to disability.

Approximately 50 participants from around the world took part in the dialogue, including persons with disabilities and people living with HIV/AIDS, as well as other policy makers, representatives of civil society, and academics. Stephen Lewis, the former United Nations Special Envoy for HIV/AIDS in Africa and co-founder of AIDS-Free World, provided the keynote address during the dialogue's opening session.

The dialogue took place through a combination of expert presentations, panels and small breakout group sessions. The detailed results of the dialogue have been outlined in a final report and disseminated via existing national and international networks on HIV/AIDS and disability. The final report is available from Health Canada.

## Dialogue overview

The three-day dialogue began with a discussion on the shared experiences and barriers to full and equitable participation in all aspects by society of persons with disabilities (PWD) and people living with HIV/AIDS (PLHIV). This included discussion of the definition of "disability", namely how inclusive a term it can be, and when it is strategic to define HIV/AIDS as a disability.

With this foundational understanding of the commonalities and differences of the experiences of PWD and PLHIV, the second day allowed for more in-depth discussion on some of the specific challenges associated with HIV/AIDS and disability. The final day of the dialogue was then used to discuss the next steps and opportunities to ensure that policy and programming agendas worldwide integrate HIV/AIDS and disability.

A number of themes emerged over the course of the dialogue, including various ways to use the Convention on the Rights of Persons with Disabilities (CRPD) as a tool for change. Although HIV/AIDS is not explicitly mentioned within the convention, Article 25 (a) may be interpreted to include HIV/AIDS as a disability. Article 25 (a) states that state parties shall: "Provide persons with disabilities with the same range, quality and standard of free or affordable health care and programmes as provided to other persons, including in the area of sexual and reproductive health and population-based public health programmes." [1]

The CRPD offers the prospect of a binding treaty and viable legal instrument that could be used to protect and promote equal rights of PWD and PLHIV, complementing (but not replacing) other human rights instruments. Dialogue participants suggested that in addition to advocating for the implementation of the CRPD and the Optional Protocol, the CRPD Committee of Experts should also be lobbied to include HIV/AIDS in guidelines for implementation, monitoring and reporting on the convention. The importance of raising awareness on the potential of the CRPD for advancing the rights of PWD and PLHIV was also highlighted.

Discussions on the intersectionality of HIV/AIDS and disability were also a major theme at the dialogue. This intersectionality manifests itself in the experiences of PWD and PLHIV:

1. PWD experience all of the risk factors associated with HIV infection and are, in fact, often at increased risk of infection because of such factors as poverty, limited access to education and increased risk of violence, including sexual assault.

2. PLHIV often develop disabilities as a result of HIV, either as a result of the disease progression or as side effects of antiretroviral treatment.

In addition to these key themes, a number of emerging issues in HIV/AIDS and disability were also discussed, identifying the challenges and gaps in policy development, programming, research, and education and/or awareness. These discussions included the basic, but fundamental, acknowledgement that PLHIV and issues related to HIV/AIDS are not adequately integrated into disability policies or programmes, and vice versa, at the international, national, or sub-national levels.

Overarching gaps were also identified regarding the availability of funding, both for programming and for research. The need for a substantial increase in the amount of research, both quantitative baseline statistics and qualitative data, was also highlighted throughout the dialogue.

Other key issues that were discussed included:

1) *The importance of disability support programmes*. In countries where social and/or disability support programmes are available, the level of support is often inadequate, resulting in PWD living in poverty or exacerbating their impoverishment. In addition, PLHIV may be excluded from benefits because of the episodic nature of their impairment.

2) *Misperceptions regarding HIV/AIDS and PWD*. There is a common misperception that PWD are sexually inactive and are unlikely to use drugs or alcohol. As a result, AIDS service organizations and health practitioners have tended to overlook the needs and risks experienced by PWD in HIV/AIDS prevention campaigns and service delivery. Even disability rights groups have tended to place a low priority on providing sexual health information and support to PWD.

3) *Mental health and HIV/AIDS*. A large proportion of PLHIV experience depression as a result of the psychosocial aspects of HIV infection. Front-line health professionals and AIDS service providers often have limited knowledge and skills related to: identifying the unique mental health problems of PLHIV; recognizing the disabling effects of these problems; and providing appropriate support and referrals.

4) *Development disabilities and HIV/AIDS*. Many people fear that providing information about sex to persons with development disabilities will increase their vulnerability, which creates barriers to access to information about sexual health and HIV/AIDS prevention. More research is needed to increase understanding of effective means for sharing sexual health information with these groups.

In response to these challenges a number of opportunities and recommended next steps emerged. These recommendations included:

• Sustaining dialogue between disability and HIV/AIDS networks, and implementing mechanisms for the exchange of research and promising practices

• Applying a disability lens to HIV/AIDS policies and programmes and, likewise, assessing disability policies and programmes to better meet the needs of PLHIV

• Building the capacity of PWD and disabled persons' organizations to facilitate meaningful involvement in HIV/AIDS activities

• Identifying regular events and meetings on either HIV/AIDS or disabilities, where the intersectionality of disability and HIV/AIDS could be profiled

• Removing barriers to full participation of PWD in HIV/AIDS prevention, treatment, care and support activities, including the promotion of healthy sexuality among PWD

• Studying the lessons learned through joint programming for HIV and tuberculosis at the international, country and grassroots level

• Promoting the inclusion of disability issues in the formal education of medical and other health care providers

• Advocating for the inclusion of PWD in national HIV/AIDS strategic plans, and inclusion of HIV/AIDS in CRPD guidelines, monitoring and reporting processes

• Developing a standard process for monitoring and reporting in order to establish baseline data and a consistent evidence base on disability and HIV/AIDS.

## Conclusion

The issue of HIV/AIDS and disability is one that has been inadequately recognized and addressed at all levels; it is an issue that requires concrete concerted action in order to respond to the needs of PWD and PLHIV. The International Policy Dialogue on HIV/AIDS and Disability, held in Ottawa in March 2009, made important headway in identifying the key gaps and some of the opportunities and next steps for action.

The dialogue also strengthened and expanded the network of people working on issues related to HIV/AIDS and disability, and enabled them to share resources, best practices, challenges and experiences. By raising the profile of these important issues and further mobilizing key partners, the dialogue provided a strengthened foundation for future action.

## Competing interests

The author declares that they have no competing interests.
